# No Effect of Microgravity and Simulated Mars Gravity on Final Bacterial Cell Concentrations on the International Space Station: Applications to Space Bioproduction

**DOI:** 10.3389/fmicb.2020.579156

**Published:** 2020-10-14

**Authors:** Rosa Santomartino, Annemiek C. Waajen, Wessel de Wit, Natasha Nicholson, Luca Parmitano, Claire-Marie Loudon, Ralf Moeller, Petra Rettberg, Felix M. Fuchs, Rob Van Houdt, Kai Finster, Ilse Coninx, Jutta Krause, Andrea Koehler, Nicol Caplin, Lobke Zuijderduijn, Valfredo Zolesi, Michele Balsamo, Alessandro Mariani, Stefano S. Pellari, Fabrizio Carubia, Giacomo Luciani, Natalie Leys, Jeannine Doswald-Winkler, Magdalena Herová, Jennifer Wadsworth, R. Craig Everroad, Bernd Rattenbacher, René Demets, Charles S. Cockell

**Affiliations:** ^1^UK Centre for Astrobiology, School of Physics and Astronomy, University of Edinburgh, Edinburgh, United Kingdom; ^2^European Space Research and Technology Centre (ESTEC), Noordwijk, Netherlands; ^3^Radiation Biology Department, German Aerospace Center (DLR), Institute of Aerospace Medicine, Cologne (Köln), Germany; ^4^Microbiology Unit, Belgian Nuclear Research Centre, SCK CEN, Mol, Belgium; ^5^Department of Biology – Microbiology, Aarhus University, Aarhus C, Denmark; ^6^Kayser Italia S.r.l., Livorno, Italy; ^7^BIOTESC, Hochschule Luzern Technik und Architektur, Hergiswil, Switzerland; ^8^Exobiology Branch, NASA Ames Research Center, Moffet Field, CA, United States

**Keywords:** microgravity (μ*g*), spaceflight, Mars gravity, BioRock, International Space Station (ISS), space microbiology, space bioproduction, bacterial cell concentration

## Abstract

Microorganisms perform countless tasks on Earth and they are expected to be essential for human space exploration. Despite the interest in the responses of bacteria to space conditions, the findings on the effects of microgravity have been contradictory, while the effects of Martian gravity are nearly unknown. We performed the ESA BioRock experiment on the International Space Station to study microbe-mineral interactions in microgravity, simulated Mars gravity and simulated Earth gravity, as well as in ground gravity controls, with three bacterial species: *Sphingomonas desiccabilis*, *Bacillus subtilis*, and *Cupriavidus metallidurans*. To our knowledge, this was the first experiment to study simulated Martian gravity on bacteria using a space platform. Here, we tested the hypothesis that different gravity regimens can influence the final cell concentrations achieved after a multi-week period in space. Despite the different sedimentation rates predicted, we found no significant differences in final cell counts and optical densities between the three gravity regimens on the ISS. This suggests that possible gravity-related effects on bacterial growth were overcome by the end of the experiment. The results indicate that microbial-supported bioproduction and life support systems can be effectively performed in space (e.g., Mars), as on Earth.

## Introduction

Microorganisms such as bacteria are the foundation of Earth’s biosphere, including the human body, and will necessarily follow humans on their journey during space exploration. Since they play many important roles in biological processes on Earth, they are also expected to be essential in space. They have been shown to pervasively inhabit space stations such as the former Mir (Novikova, 2004) and the International Space Station (ISS) (Ichijo et al., 2016; Mora et al., 2019; Sielaff et al., 2019), with both negative effects and positive uses. Potential roles of microorganisms in space will include manufacturing (Menezes et al., 2015a, b), as building blocks of ecosystems (Gòdia et al., 2002), and in biomining on celestial bodies (Schippers et al., 2013; Reed et al., 2016; Jerez, 2017). Space-based research on microorganisms has been performed for the bioproduction of antibiotics (Klaus et al., 2001; Benoit et al., 2006), other secondary metabolites (Huang et al., 2018) and vaccines (Ruttley et al., 2009) for terrestrial consumption. In addition to being useful, they also present challenges, e.g., through the formation of corrosive biofilms (Gu et al., 1998) and altered virulence in space (Wilson et al., 2007; Rosenzweig and Chopra, 2012).

The term microgravity (μ*g*) is commonly used to describe a gravitational accelerations smaller than 10^–2^ × *g* and close to 10^–6^ × *g* (Horneck et al., 2010; Zea et al., 2017; Huang et al., 2018), and it is experienced for instance by objects orbiting around a celestial body in space, such as satellites in Low Earth Orbit (LEO). The effects of microgravity on eukaryotic multicellular organisms has been extensively reported for plants (Gòdia et al., 2002; Matía et al., 2010; Kiss et al., 2012; Paul et al., 2013; Zabel et al., 2016), animals (Wassersug et al., 2005; Bart et al., 2019; Maupin et al., 2019; Tahimic et al., 2019) and humans (Cavanagh et al., 2005; Van Ombergen et al., 2017; Axpe et al., 2020). Results on eukaryotic microorganisms such as *Euglena* and *Paramecium* spp. demonstrated the ability to respond to gravity (Hader, 1996; Hader et al., 1996; Hemmersbach et al., 2001). The effects of microgravity on bacterial growth, in contrast, have been controversial and inconclusively discussed (Leys et al., 2004; Taylor, 2015), despite the enormous interest in their physiological responses to space conditions. Even less studied are the effects of partial gravity conditions, such as lunar (0.16 × *g*) or Martian (0.38 × *g*) gravity, on bacterial growth (Hemmersbach et al., 1996; Santosh et al., 2011; McCutcheon et al., 2016).

The majority of the data on bacterial growth in microgravity are the results of separate projects with necessarily diverse experimental designs and a limited number of replicate samples, and overall they addressed various endpoints for distinct scientific questions (Pollard, 1965; Bouloc and D’Ari, 1991; Gasset et al., 1994; Kacena et al., 1997, 1999b; Kacena and Todd, 1997; Klaus et al., 1997; Baker et al., 2004; Nickerson et al., 2004; Horneck et al., 2010; Mulders et al., 2011; Kim et al., 2013a; Zea et al., 2016, 2017; Huang et al., 2018; Milojevic and Weckwerth, 2020; Nicholson and Ricco, 2020). Consequently, it is difficult to draw generalizable conclusions on the effects of gravity on bacterial cell growth.

Some studies concluded that cells with a diameter of less than 10 μm cannot sense gravity directly (Pollard, 1965; Horneck et al., 2010; Zea et al., 2017), and the response observed in a low-gravity experiment was ascribed to indirect effects on the local liquid microenvironment surrounding the cell (Klaus et al., 1997; Zea et al., 2016). *Escherichia coli* ATCC 4157 cells showed an increased final cell concentration in microgravity compared to the ground, which was attributed to factors such as upregulation of starvation-related and carbon source-uptake genes (Zea et al., 2016). Nevertheless, other studies using different *E. coli* strains did not confirm this observation after space flight (Bouloc and D’Ari, 1991; Gasset et al., 1994), while an experiment in simulated microgravity showed medium-dependent changes (Baker et al., 2004). Growth of *Bacillus subtilis* in microgravity produced inconclusive results as well (Kacena et al., 1997, 1999b; Kacena and Todd, 1997; Nicholson and Ricco, 2020). Similar discrepancies were reported for other bacterial species (Kim et al., 2013a) and for eukaryotic microorganisms (Mulders et al., 2011). A summary of the data on microbial growth in microgravity can be found in Huang et al. (2018).

**FIGURE 1:**
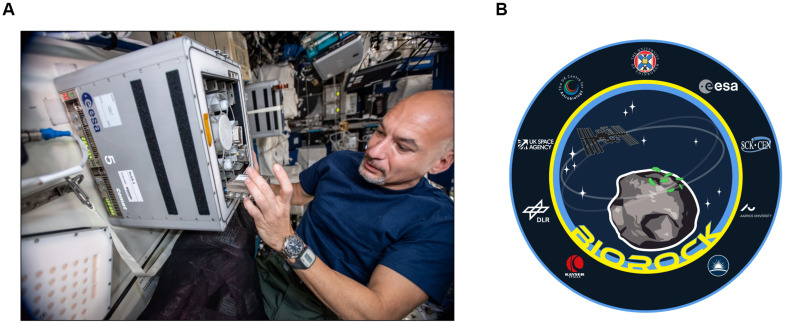
Astronaut Luca Parmitano working on BioRock. **(A)** Luca Parmitano installs the hardware into the KUBIK incubator located in the Columbus module onboard the ISS. Image credit: ESA. **(B)** The official BioRock logo (created by Hadrien Jouet and Mauro Manzo).

In response to a flight opportunity offered by the European Space Agency (ESA), we conducted the BioRock experiment (Loudon et al., 2018) onboard the ISS with the general aim to advance the knowledge on bacterial responses to microgravity (μ*g*), simulated Mars (Mars *g*) and Earth (Flight 1-*g*) gravities, with a view to microbe-mineral interaction and its potential roles in extraterrestrial life support systems, e.g., *in situ* Resource Utilization (ISRU), biomining and soil formation from planetary regolith (Cockell, 2010; Olsson-Francis et al., 2010; Zaets et al., 2011; Menezes et al., 2015a) (Figure 1).

We selected three bacterial strains with demonstrated evidence for their ability to interact with rock surfaces or soil, bioleach, grow on surfaces and resist desiccation: *Sphingomonas desiccabilis* CP1D, *Bacillus subtilis* NCIB 3610 and *Cupriavidus metallidurans* CH34. As BioRock had the rare opportunity to study multiple microorganisms in the same set of experimental conditions, one part of this experiment was to test the hypothesis that the gravity condition influences the final cell concentration of the bacterial cultures after a 3-week period. This timeframe was chosen as a point in stationary phase that gives enough time to create microbe-mineral interactions, but not yet in death phase to lead to cell degradation (Loudon et al., 2018). Despite the diverse gravity-dependent phenomena involved, we reported no effect of gravity on the final cell concentrations of the three different bacterial species. We discuss the importance of these results for the future of human space exploration (Horneck et al., 2010), as the duration and ambition for spaceflight missions expands, as well as for terrestrial applications.

## Materials and Methods

### Bacterial Strains, Medium and Fixative

For this experiment, we selected three bacterial species with demonstrated evidence for their ability to grow on and interact with rock surfaces or soil, bioleach elements and resist dessication (Loudon et al., 2018): (i) *Sphingomonas desiccabilis* CP1D (DSM 16792, University of Edinburgh), a Gram-negative, non-motile and non-spore-forming α-proteobacterium (phylum Proteobacteria) (Reddy and Garcia-Pichel, 2007). *S. desiccabilis* was first isolated in the Colorado plateau from soil crusts (Stevens et al., 2019). It was selected for its high resistance to desiccation and for its natural presence in desert crust environments (Reddy and Garcia-Pichel, 2007; Stevens et al., 2019). (ii) *Bacillus subtilis* NCIB 3610 (DSM 10) [German Aerospace Center (DLR) Cologne, Germany] (Nye et al., 2017), a Gram-positive, motile and spore-forming bacillus (phylum Firmicutes). *B. subtilis* is a very well characterized model organism and has been widely used in space experiments (Kacena et al., 1999b; Fajardo-Cavazos et al., 2018; Morrison et al., 2019; Nicholson and Ricco, 2020). It can survive in harsh environments and grow on rock substrates (Song et al., 2007). Moreover, it was found as a common contaminant on the ISS (Nickerson et al., 2004); (iii) *Cupriavidus metallidurans* CH34 (SCK CEN, Belgium), a Gram-negative, non-spore-forming, motile β-proteobacterium (phylum Proteobacteria) (Janssen et al., 2010). Strains of this species have been isolated from rocks and metal-contaminated environments (Janssen et al., 2010; Olsson-Francis et al., 2010; Bryce et al., 2016; Vandecraen et al., 2016), and it has been previously isolated from ISS and used in space experiments (Leys et al., 2009; Monsieurs et al., 2014; Byloos et al., 2017). The main bacterial characteristics of the three strains are summarized (Supplementary Table 1).

All organisms were grown in an identical medium which was approved for spaceflight as a compromise to allow the growth of the three species (Loudon et al., 2018). Five milliliters of a 50% v/v solution of R2A growth medium (from now on referred to as 50% R2A) were used for each sample (Reasoner and Geldreich, 1985), containing (g L^–1^): yeast extract, 0.25; peptone, 0.25; casamino acids, 0.25; glucose, 0.25; soluble starch, 0.25; Na-pyruvate, 0.15; K_2_HPO_4_, 0.15; MgSO_4_⋅7H_2_O, 0.025, adjusted to a final pH of 7.2.

The fixative chosen to stop cell growth at the end of the experiment was NOTOXhisto (Scientific Device Laboratory), a formalin-free ethanol-based solution previously tested for its ability to stop cell growth of the selected bacteria, for its biocompatibility with the hardware and the safety requirements on the ISS (Loudon et al., 2018). One milliliter of fixative was used for each sample, with a final volume ratio of 1:5 fixative-medium.

### Pre-Flight Sample Preparation

Single strain cultures of each bacterial species were desiccated on a sterile basalt slide cut from a rock specimen, taken near Gufunes, Reykjavik in Iceland (64°08’22.18”N, 21°47’21.27”W). Rock sterilization was performed by dry-heat sterilization in a hot air oven for 4–5 h at 250°C. Negative controls were sterile basalt slides without cell inoculation.

Each bacterial population was treated differently for sample preparation. This affected the initial cell numbers on the basalt slides, which was different for each bacterial strain, but it was necessary to ensure optimal storage conditions for each organism during pre-activation.

For *S. desiccabilis*, an overnight culture of the strain was grown in R2A broth at 20–22°C. When the culture reached stationary phase (OD_600_ = 0.88 ± 0.09, corresponding to approximately 1 × 10^9^ CFU mL^−1^), each basalt slide was soaked in 1 mL of the bacterial culture and samples were air-dried at room temperature in a laminar flow hood under sterile conditions. The protocol for *B. subtilis* spore production and sample preparation has been previously described (Fuchs et al., 2017). Ten microliters of a thoroughly mixed ∼1 × 10^8^ spores mL^–1^ solution were used as inoculum for each basalt slide (resulting in ca. 1 × 10^6^ spores per slide) and air-dried at room temperature in a laminar flow hood under sterile conditions. *C. metallidurans* samples were prepared by using a freeze-drying protocol (Belgian Co-ordinated Collection of Micro-organisms, BCCM). Cells were cultured on solid Tryptone Soya Agar (Oxoid CM0131, BCCM), harvested and suspended in a lyoprotectant [sterile horse serum supplemented with 7.5% w/v trehalose (final concentration) and broth medium n°2 (final concentration: 625 mg L^–1^; Oxoid CM0131, BCCM)]. Basalt slides were submerged in 30 mL of a bacterial suspension and gently shaken overnight. Basalt slides were then inserted on a pre-cooled shelf at −50°C, followed by a freezing phase for 90 min at a shelf temperature of −50°C. Primary drying was performed at −18°C and chamber pressure of 400 mTorr, secondary drying was performed at 20°C and a chamber pressure below 10 mTorr. After the procedure, each basalt slide contained approximately 1 × 10^9^ CFU mL^–1^ of *C. metallidurans*.

Samples were stored at room temperature until integration in the Experimental Units (EUs). Samples in each gravity condition, including the negative control, were present in triplicate.

### Setup of the Flight Experiment

**FIGURE 2:**
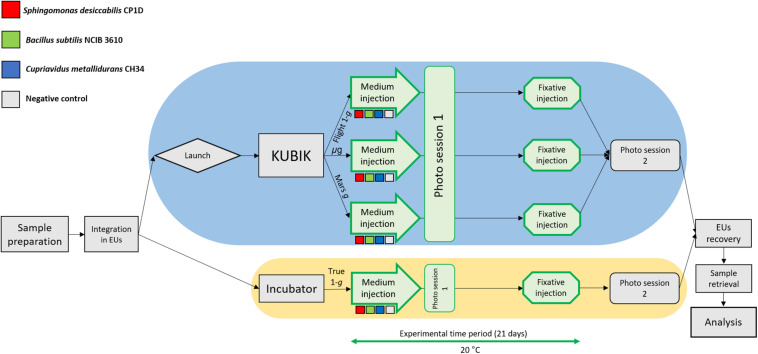
Flow diagram of the BioRock experiment. Three bacterial species were prepared for the experiment: *S. desiccabilis* (red squares), *B. subtilis* (green squares), and *C. metallidurans* (blue squares). Negative controls (gray squares) represented sterile basalt slides. After integration in the experimental units (EUs), the EUs were either launched to the ISS (blue oval), where they were installed in KUBIK incubators and subjected to microgravity (μ*g*), simulated Mars gravity (Mars *g*) or simulated Earth gravity (Flight-1*g*), or kept for incubation on Earth for the ground gravity control (True-1*g*, yellow oval). Steps in green were part of the experimental time period (21 days). Storage passages were omitted for brevity. Each experimental set was represented in triplicate.

A diagram of the experimental setup is presented in Figure 2. After preparation of the hardware, samples, medium and fixative were integrated under aseptic conditions into the designated flight Experiment Unit (EU) before the launch. Each EU was composed by two BioMining Reactors (BMRs). A BMR represents a culture chamber of 15 × 14 × 23.2 mm that can contain 6 mL of liquid volume after hardware activation and medium injection, delimited by the basalt slide (after sample integration) on one side, and a semipermeable membrane on the remaining five sides. The semi-permeable silicon membrane allowed gas diffusion. Each BMR is connected to a 5 mL medium reservoir and a 1 mL fixative reservoir, which were activated at the appropriate time (Supplementary Figure 1). The EUs were also equipped with temperature loggers (installed in four EUs, Signatrol SL52T sensors, Signatrol, United Kingdom) and accelerometers (in all EUs on ISS), that allowed the measurement of the acceleration profiles over the experiment (Supplementary Table 2). For a complete description of the EU, refer to Loudon et al. (2018). A total of 36 samples in 18 EUs for the flight experiment and 12 samples in 6 EUs for the ground control were prepared. After integration, the 18 flight EUs were stored at room temperature and sent to the ISS on board of a Space X Falcon-9 rocket (CSR-18 mission) on July 25th, 2019, from the NASA Kennedy Space Centre, Merritt Island, FL, United States. On arrival to the ISS on July 27th, the samples were stored at 2–8°C in an onboard refrigerator until being installed into the two KUBIK (ESA) incubators aboard the station, previously set to 20°C. Once in KUBIKs, the EUs were let to adjust to the incubation temperature for one hour. Then, the automatic timeline of the EUs was activated and medium (5 mL) was injected in consecutive manner to each culture chamber (18 BMRs per KUBIK) within 1.5 h. The medium injection could not be done at once for all BMRs due to KUBIKs power limitations. All samples were then removed from KUBIK 5, first image acquisition was performed, and the EUs were re-installed into the KUBIK 5 (EUs were out of KUBIK for circa 40 min) and this latter was reactivated. The same procedure was then performed for KUBIK 6 (EUs were out of KUBIK for circa 20 min). All crew activities were performed by Luca Parmitano. The KUBIK 5 centrifuge was set to 1.2 × *g* to simulate Earth gravity (Flight 1-*g*), KUBIK 6 was set to 0.5 × *g* to simulate Mars gravity (Mars *g*). These settings were chosen to obtain a final gravity acceleration of 1 × *g* and 0.4 × *g*, respectively, as the geometry of the BMR locates the sample at a shorter radius than the reference radius, meaning that the acceleration experienced by the sample is lower than the set-point. Notably, a strict Martian gravity of 0.38 × *g* was not settable on the KUBIK, as the setting allows gravity increments of 0.1 × *g*. Samples grew for 21 days at 20.16°C (temperature loggers). At the end of the experiment, 1 mL of fixative was injected consecutively in the culture chambers within 1.5 h (on August the 20th, 2019). A second photo session was performed and hardware were then cold stored at 2.1–7.1°C (logged data). On orbit, the EUs were stored in the MELFI hardware, and by packaging in a “Double Coldbag” provided by NASA for download, with SpaceX CRS-18 (same vehicle as for upload). Samples were retrieved at NASA Ames Research Centre, Mountain View, CA, United States together with the ground controls.

Prior to the space experiment, the ground control units were shipped in cold stowage (logged data 3.58–4.54°C) to NASA Ames Research Centre. The procedure started two days after the start of the space experiment and the ground experiment was subject to analog procedures and conditions to those occurring to the flight hardware. The six EUs were incubated at 20°C in a laboratory incubator (Percival E30BHO incubator) and attached to a power system to activate the hardware. The temperature loggers recorded a constant temperature of 20.62°C. Medium was injected in a similar manner as in KUBIK onboard the ISS using the KISS (KUBIK Interface Simulation) power system, the first photo session was performed (duration circa 20 min), and the experiment was conducted for a period of 21 days. After fixative injection, photo session 2 was performed (duration circa 20 min) and EUs were stored at 3.06°C (logged data) until sample retrieval. We refer to these samples as True 1-*g*.

Samples were recovered, separating the culture liquids, the basalt slides and the membranes. The liquid cultures analyzed in this work were stored at 2–8°C until analysis. Medium injection was successful in all flight and ground samples. Fixative injection was successful for all the flight BMRs, but four ground BMRs (containing *B. subtilis*, *C. metallidurans*, *C. metallidurans*, and a negative control, respectively) suffered from fixative injection failure. In these cases, 1 mL of NOTOXhisto was added to the liquid samples immediately after sample recovering and before storage. All samples were shipped back to the University of Edinburgh in cold stowage by Altech Space (Torino, Italy) and analyzed.

### Images Acquisition (Photo Session) Setup

The photographs onboard the ISS were taken in microgravity with a Nikon D5 camera with a 105 mm lens with the following settings: F/10, ISO-1000. EUs were setup in a dedicated custom-made sample holder, which was fixed on the Maintenance Work Area (MWA; Supplementary Figure 2). The camera was fixed on the opposite side of the MWA. A work light was set up next to the camera to minimize reflection on the transparent window of the EU. The photo session for the ground control (True 1-*g*) was performed with a POT-LX1 camera with the following settings: F/1.8, ISO-80. In this case, no sample holder or particular working area was necessary.

### Determination of Final Cell Numbers

Cell population after sample retrieval was assessed on the liquid samples by two methods: direct cell counting and optical density.

Direct cell counting was used to determine the cell concentration for each sample by fluorescence microscopy. The cell suspension was diluted in R2A medium and stained with SYBR Gold (Invitrogen by Thermo Fisher Scientific, 1:10,000 diluted). Samples were vortexed for 30 s and incubated in the dark for 30 min. After vortexing the samples again for 30 s, samples were filtered on black polycarbonate Nuclepore Track-Etched Membranes with 0.2 μm pore size (Whatman) and visualized by fluorescence microscope (Leica DM 4000 B, Leica Microsystems). Cell numbers were counted for 50 fields of view at 100× magnification. When cell concentration was too low to ensure an appropriate number of cells per field of view, cell suspensions were concentrated, or 100 fields of view were counted.

For optical density analysis, an absorbance microplate reader (BMG Labtech) set to a wavelength of 600 nm was used.

Statistical comparisons were performed using a one-way ANOVA analysis, followed by a *post hoc* Tukey test to determine whether significant differences existed between different experimental groups (R version 3.6.3). Significance was set at *p*-values of ≤ 0.05.

### Spore Enumeration

Spore enumeration was determined from the liquid samples of *B. subtilis* after spaceflight. A colony formation unit (CFU) assay was used. Total cell count and spore counts (after heating for 15 min at 80°C; 50 μL sample volume per test in triplicates) were determined from appropriate dilutions in sterile buffered phosphate saline (0.7% w/v Na_2_HPO_4_ × 2H_2_O, 0.3% w/v KH_2_PO_4_, 0.4% w/v NaCl pH 7.5) as colony-forming ability after incubation overnight at 37°C on nutrient broth agar plates (Difco, Detroit, MI, United States). The data shown are expressed as means ± standard deviations. The results were compared statistically using Student’s *t* test (SigmaPlot13, Systat Software). Values were analyzed in multigroup pairwise combinations, and differences with *p* values ≤0.05 were considered statistically significant.

### Ground Experiment: Cell Growth in the Presence of the Basalt Rock

Fresh pre-cultures of the three different organisms were grown overnight until reaching stationary phase. Then, optical density (λ = 600 nm) was measured, and an appropriate amount of each culture was diluted in 50% R2A in order to reach a starting OD_600_ of 0.050–0.071. Five milliliters of each bacterial starting solution were then inoculated into a 6-well plate, with and without the presence of a basalt slide, all in triplicates. A 6-well plate for each time-point and for each organism was prepared, plus the same number of samples with fresh 50% R2A for the negative controls. All the samples were kept at 20°C, and OD_600_ was measured at each time point (day 0, 1, 4, 7, 14, and 21). Contrary to the space experiment, vegetative cells were used in this experiment for all the three organisms, included *B. subtilis*, without previous desiccation on the basalt slide.

### Calculations of Cell Sedimentation Rates and Diffusion Under Microgravity, Mars and Earth Gravity

An estimation of the velocity of sedimentation of a single cell (*v*_*cell*_) under the artificial Mars and Earth gravity regimens was calculated using the Navier-Stokes Eq. (1) (Todd, 1989):

(1) $$\upsilon_{c\,e\,l\,l}=\frac{2\times \left(\rho -\rho_{0}\right)\times a^{2}\times g}{9\times \eta }$$

where *ρ*_o_ is the fluid density (g cm^–3^), *ρ* is the density of the particle (in this case the cell, g cm^–3^), *a* is the effective Stokes radius of the cell (m), *g* is the gravitational acceleration (m s^–2^), and η is the fluid viscosity (kg h^–1^ m^–1^).

The distance a given cell will sediment (*d*_sed_) within the culture chamber in a given time *t* (s) is given by:

(2) $$d_{s\,e\,d}=\upsilon_{c\,e\,l\,l}\times t$$

We also calculated the distance that a cell would cover by Brownian motion over a given time (*x*), which can be calculated using the Eq. (3):

(3) $${<x>}^{2}=2\times D\times t$$

Where <*x*>^2^ is the root-mean-square diffusion distance traveled (cm^2^), *D* is the diffusion coefficient (cm^2^ s^–1^) and *t* is the time (s).

The diffusion coefficient *D* was calculated using the Eq. (4) (Klaus et al., 1997):

(4) $$D=\frac{\left(k_{B}\times T\right)}{\left(6\times \pi \times \eta \times a\right)}$$

Where *k*_B_ is the Boltzmann constant (1.38 × 10^–23^ J mol^–1^) and *T* is the temperature, which is 295.13 K in our experiments. The diffusion coefficient using Eq. (4) was 4.02 × 10^–13^ m^2^ s^–1^.

For these calculations, we took an effective Stokes radius of 6.0 × 10^–7^ m (using a cell diameter of 1.2 μm), a cell density of 1.08 g cm^–3^, and a fluid density similar to water of 1.01 g cm^–3^. The medium viscosity was assumed to be similar to water (1.002 mPa s at 20°C).

The KUBIK centrifuges delivered 1 × *g* (Flight 1 × *g*) and 0.4 × *g* (Mars *g*) at the top surface of the basalt slides, with 1.3-fold increase of acceleration at the membrane surface, leading to gravity accelerations of 1.3 × *g* and 0.5 × *g* at the membrane surface, respectively. Because sedimentation at a given gravity would imply sedimentation at higher gravity accelerations, we used the lowest experimental values (9.81 and 3.67 m s^–2^ for Earth and Mars, respectively) for these calculations. For the two microgravity regimens, we used 9.81 × 10^–2^ m s^–2^ and 9.81 × 10^–6^ m s^–2^ for 1 × 10^–2^ *g* and 1 × 10^–6^ *g*, respectively.

## Results

### Final Cell Concentration and Optical Density

**FIGURE 3:**
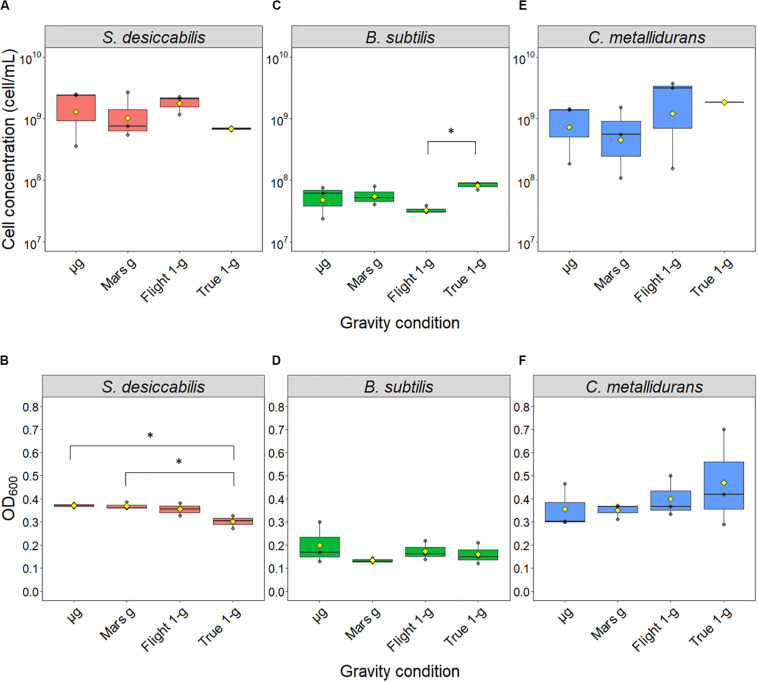
Final cell concentrations obtained after spaceflight. Boxplots of the direct cell counts (upper panel) and optical densities at a wavelength of 600 nm (lower panel) distributions are shown for *S. desiccabilis* **(A, B)**, *B. subtilis* **(C, D)**, and *C. metallidurans* **(E, F)**, respectively. Each dot represents a single measurement (*n* = 3). Yellow diamonds represent mean values. The horizontal bar indicates the median and boxes represents the 25^th^ to 75^th^ percentile. Asterisks (^∗^) indicate statistically significant differences (overall α = 0.05, Tukey honestly significant difference correction).

The samples launched to the ISS were exposed to three different gravity regimens (microgravity, μ*g*; simulated Martian gravity, Mars *g*; simulated terrestrial gravity, Flight 1-*g*), while the reference experiment was conducted on Earth at 1 × *g* (ground control samples, True 1 × *g*) (Figure 2). The cultures were grown for 21 days in the presence of a basalt slide. For *S. desiccabilis* (Figures 3A,B), ANOVA on final cell counts showed no difference between the gravity regimens (0.4–2.7 × 10^9^ cell mL^–1^, *F*(3, 8) = 1.052, *p*-value = 0.421; Figure 3A). ANOVA on optical density measurements confirmed the lack of difference between the three gravity conditions tested in space (μ*g*, Mars *g* and Flight 1 × *g*; Figure 3B). However, the optical density of True-1*g* samples was significantly lower compared to samples subject to μ*g* and Mars *g* [*F*(3,8) = 7.148, *p*-value = 0.0119, *post hoc* Tukey test *p*-values between μ*g* and True-1*g* = 0.016, and between Mars *g* and True-1*g* = 0.018; Figure 3B]. This is in contrast to the lack of significant difference observed for final cell counts. Significant differences in optical density were observed neither between μ*g* and Flight-1*g*, nor between Mars *g* and Flight-1*g* (both *p* values > 0.05; Figure 3B). Cultures grown at simulated (Flight-1*g*) and real (True-1*g*) Earth gravity showed no significant difference (*p*-value = 0.06; Figure 3B) in optical density. This suggested that the difference in optical densities between the μ*g* or Mars *g* conditions and the True-1*g* could not be explained by gravity alone and may have been subject to additional influencing factors.

*Bacillus subtilis* (Figures 3C,D) showed no significant difference in final cell counts [ANOVA: *F*(3,8) = 3.806, *p*-value = 0.058] and optical densities [ANOVA: *F*(3,8) = 0.776, *p*-value = 0.54]. No difference was observed between space and ground-based samples (2.4–8.9 × 10^7^ cell mL^–1^, *post hoc* Tukey test *p* values > 0.05; Figure 3C) with the exception of a lower final cell counting of cultures grown under simulated Flight-1*g* compared with True-1*g* (*post hoc* Tukey test *p*-value = 0.04). We note that, in contrast to the other bacterial strains tested in this space experiment, *B. subtilis* cultures were initiated as spores, which had to germinate and form highly motile vegetative cells before they could multiply (Setlow et al., 2017). Furthermore, the use of 50% R2A as a necessary compromise for the three microorganisms could have led to lower cell concentrations compared to media with higher organic concentrations.

Similar to *B. subtilis*, the final cell counts of *C. metallidurans* cultures (Figures 3E,F) were not significantly influenced by gravity conditions, as indicated by the results from both analytic methods [ANOVA on cell count data: *F*(3,8) = 1.409, *p*-value = 0.309; ANOVA on optical density data: *F*(3,8) = 0.595, *p*-value = 0.636]. This is probably due to a large variability, which resulted in lack of statistically significant differences even when the average values for cell concentration differed in order of magnitude (∼10^8^ cell mL^–1^ for cells in Mars *g*, ∼10^9^ cell mL^–1^ for μ*g*, Flight-1*g* and True-1*g*). The cause of this variability is unknown. It could have been produced by a different initial cell number or a difference in growth phase stage (stationary phase, decline/death phase) elicited by nutrient and oxygen availability.

### Spore Enumeration for *B. subtilis* Samples

*Bacillus subtilis* was the only spore-forming bacterium used in the BioRock experiment. The spore numbers from *B. subtilis* liquid samples were counted.

The initial number of spores inoculated on each basalt slide was 1 × 10^6^ spores/slide. The results (Table 1) showed effective germination of the initial spores, growth as vegetative cells and sporulation again in space, thus going through at least one complete life cycle. Spore formation during spaceflight was not affected by the gravity regime (*p* value > 0.05).

**TABLE 1:** Enumeration of *B. subtilis* spores in liquid samples. CFU assay data are shown as averages ± standard deviation (*n* = 9). Each sample was tested in triplicate. The ratio between the number of spores and the total cell counts is shown and expressed as a percentage.

*g* regime	Total cells count (CFU mL^–1^)	Spore count (CFU mL^–1^)	Spores/total cells (%)
μ*g*	2.4 ± 0.5 × 10^7^	2.5 ± 0.2 × 10^6^	10.5 ± 1.2
Mars *g*	2.0 ± 0.2 × 10^7^	1.8 ± 0.5 × 10^6^	8.5 ± 1.5
Flight 1-*g*	2.5 ± 0.3 × 10^7^	1.7 ± 0.4 × 10^6^	6.9 ± 1.8
True 1-*g*	2.5 ± 0.8 × 10^7^	2.0 ± 0.6 × 10^6^	8.0 ± 2.1

### Influence of the Basalt Slide on Cell Growth

As the primary objective of the BioRock experiment was to test the interaction of the microorganisms with minerals in different gravity regimens, the cultures were grown in the presence of a basalt slide. To determine whether the presence of the basalt slide influenced the final cell concentration, a ground experiment was performed. The three bacterial strains used in the BioRock experiment were grown in 50% R2A broth in the presence or absence of a basalt slide. Cell-free 50% R2A medium, supplemented with or without basalt, was used as negative control.

**FIGURE 4:**
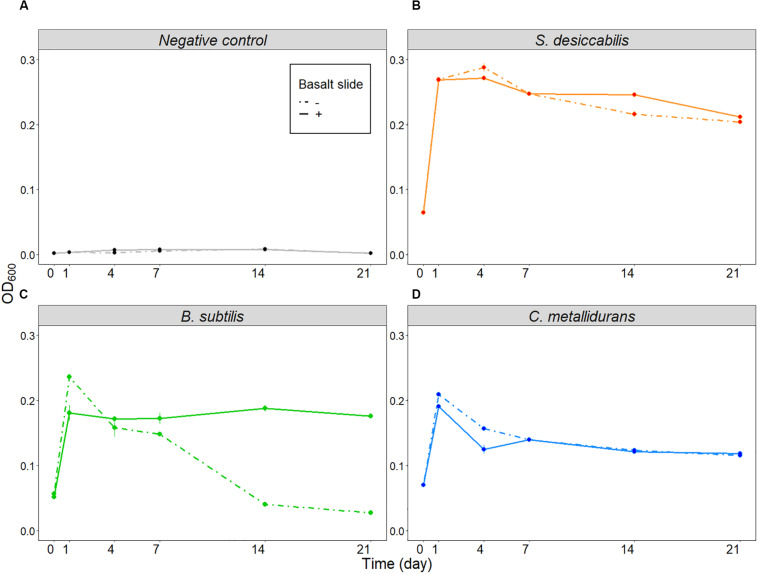
Influence of basalt slide on cell growth (ground experiment). Average optical densities for the three organisms, plus the negative control, over 21 days in the presence or absence of the basalt slide. **(A)** Shows the results for the negative controls, **(B)** for *S. desiccabilis*, **(C)** for *B. subtilis*, and **(D)** for *C. metallidurans*. Dotted lines represent data from cell cultures grown in the absence of the basalt slide, continuous lines represent growth in the presence of the basalt slide. Mean values from three independent experiments are plotted at each time-point (*n* = 3). Error bars represent standard errors.

The negative control (Figure 4A) was used to demonstrate that changes in optical density were not caused by the presence of the basalt slide. The final optical density of *S. desiccabilis* (Figure 4B) and *C. metallidurans* (Figure 4D) was not influenced by the presence of the basalt slide *per se*, although some fluctuations were observed throughout the experimental period. Interestingly, optical density of *B. subtilis* cultures depended on the presence of the basalt slide for sustained growth (Figure 4C). While a higher value was measured after one day of growth in the absence of the rock, the overall optical density was strongly reduced after 14 days, in comparison to the cultures that were grown in the presence of a basalt slide. The CFU assay performed on *B. subtilis* cultures after the 21^st^ day revealed a cell concentration of 1.97 ± 0.51 × 10^9^ CFU mL^–1^ (mean ± SE) for the samples grown in the presence of the basalt slide, and 2.03 ± 0.22 × 10^8^ CFU mL^–1^ when the slide was absent. Hence, the lower optical density measured for the cultures in the absence of a basalt slide could be partially explained by entrance in a quiescent state and spore formation. We note differences in final cell concentrations of *B. subtilis* in these experiments compared to the True 1-*g* samples. This might be accounted for by technical differences, such as the growth in 6-well plate and the use of a fresh overnight culture of vegetative cells to start the experiment, with no period of prior desiccation. Taken together, our data demonstrated that the presence of the basalt rock in the culture did not hinder bacterial growth, on the contrary it demonstrated the potential to enhance cell growth and survival.

### Cell Sedimentation and Diffusion Under Microgravity, Mars and Earth Gravity

**FIGURE 5:**
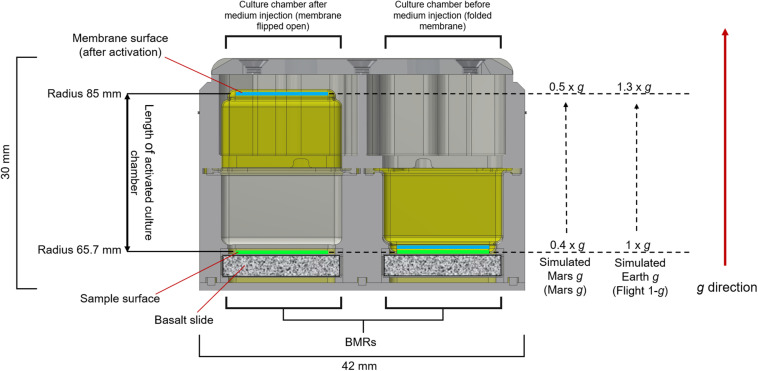
Gravity levels provided by the KUBIK inside the BMR chamber. The image shows a cross-section of the Experimental Unit (EU) before (right BMR) and after (left BMR) activation by medium injection. The membranes are colored in yellow. 1 × *g* and 0.4 × *g* were delivered at the top surface of the basalt slides (sample surface). When the membrane flips (left BMR), following medium injection in the chamber, the radius of the culture chambers ranges from 65.7 mm to approx. 85 mm. In the KUBIK incubators, the direction of the gravity vector imposed on the EU goes from the basalt slide toward the membrane surface. This causes a 1.3-fold increase of gravity across the culture chamber, leading to a gravity acceleration on the membrane surface of 1.3 × *g* (Flight 1-*g*) and 0.5 × *g* (Mars *g*).

Sedimentation of cells has relevant effects on a liquid culture (Benoit and Klaus, 2005); however, it does not occur in microgravity (Klaus et al., 1997). To estimate whether we would expect cell sedimentation under Martian and terrestrial gravity regimens over the timeframe of our experiment, we calculated the velocity of sedimentation of a single cell (*v*_*cell*_). Calculations with two different microgravity regimens were added to demonstrate the negligibility of sedimentation in this gravity condition for the timeframe of our experiment. For the microgravity calculation, we used both the upper limit (1 × 10^–2^ × *g*) and one commonly used value when defining microgravity (1 × 10^–6^ × *g*). Cell sedimentation velocity (*v*_*cell*_) values for Earth and Mars gravity were 5.48 × 10^–8^ and 2.05 × 10^–8^ m s^–1^ (Table 2, column III), or 4.74 and 1.77 mm day^–1^, respectively (Table 2, column IV). Considering an estimated maximum length of the culture chamber of 2.3 cm (the distance from the surface of the basalt rock to the outer membrane, Figure 5), it would have taken a cell 4.86 and 12.98 days, in Earth and Mars gravity respectively, to settle on the outer membrane (Table 2, column VI). A bacterial cell would have taken at least 486 days to settle in the culture chamber in microgravity (Table 2, column VI).

**TABLE 2:** Values for the calculated sedimentation rates. Cell sedimentation velocity (*v*_*cell*_) was calculated for Earth *g*, Mars *g* and microgravity (columns I–II). For this latter we used two different values (1×10^−2^×*g* and 1×10^−^6×*g*). Different units are shown to underline the estimated cell sedimentation rate in the culture chamber over the BioRock experiment (columns III–VI). The distance a cell would travel by random Brownian movements during the time necessary to settle in the culture chamber (column VI) is reported in column VII.

I	II	III	IV	V	VI	VII

Gravity condition	Gravity regime (× *g*)	*v*_*cell*_ (m s^–1^)	*v*_*cell*_ (mm day^–1^)	Distance in 21 days (cm)	Time to travel 2.3 cm (day)	Distance by Brownian diffusion with *t* from VI (mm)
Earth *g*	1	5.48 × 10^–8^	4.74	9.95	4.86	0.55
Mars *g*	0.4	2.05 × 10^–8^	1.77	3.72	12.98	0.90
μ*g* (upper value)	1 × 10^–2^	5.48 × 10^–10^	4.74 × 10^–2^	9.95 × 10^–2^	4.86 × 10^2^	5.49
μ*g* (typical value)	1 × 10^–6^	5.48 × 10^–14^	4.74 × 10^–6^	9.95 × 10^–6^	4.86 × 10^6^	5.49 × 10^2^

By contrast, the diffusion rate is independent of gravity (Klaus et al., 1997). The calculations showed that the distance traveled by a cell-size object due to random Brownian movement would have been only 0.25 mm in one day, and 1.14 mm in 21 days. Therefore, during the time it would have taken for a cell to sediment from one end of the culture chamber to the other (2.3 cm, Table 2, column VI) in Earth and Mars gravity conditions, a cell would have randomly traveled only 0.55 and 0.90 mm respectively (Table 2, column VII).

It must be noted that these calculations are necessarily estimates, e.g., assuming the cells are spherical shaped. However, due to the dependency of cell shape upon their growth stage, nutritional availability and space conditions (Zea et al., 2017), calculations resulting from modeling the organisms’ shape would have been equally approximated. Motility was not considered here. Nevertheless, these calculations allowed us to estimate the occurrence of cell sedimentation in the timeframe of the experiment.

### Cell Aggregation Revealed by Post-Fixation Photo Session

**FIGURE 6:**
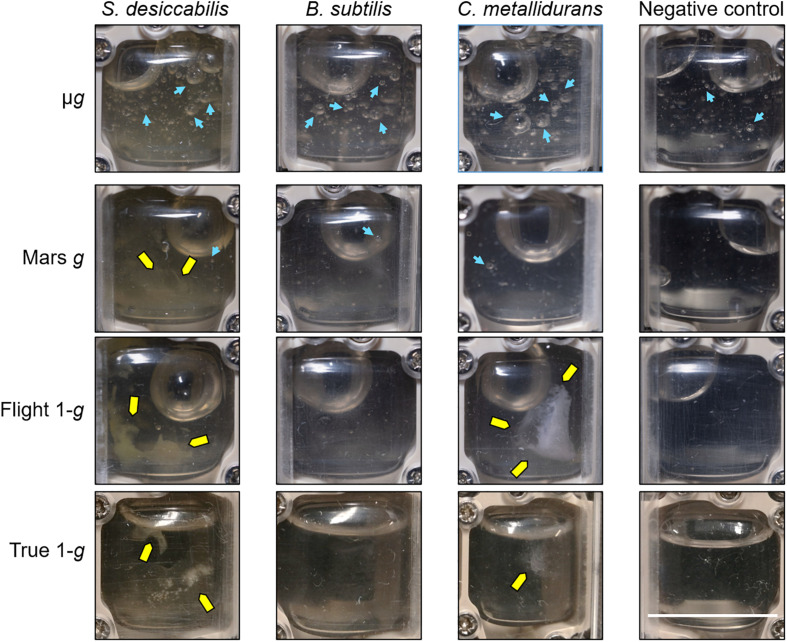
Images from the second (post-fixation) photo session onboard the ISS. Representative photos of the individual culture chambers after three weeks of growth and fixative injection. Each image shows the culture chamber of a single BioMining Reactor (BMR). Pictures of the samples in microgravity (μ*g*), Mars gravity (Mars *g*) and terrestrial gravity (Flight-1*g*) were taken during the spaceflight (in microgravity), while pictures of the ground controls (True-1*g*) were taken at NASA Ames Research Center (at 1 × *g*). Yellow arrows indicate aggregates. Blue arrows indicate some of the small gas inclusions (bubbles) discussed in the main text. The scale bar has been measured on the shorter side of the BMR and corresponds to ∼14 mm.

Two series of photos (photo sessions) of the Experimental Units (EUs) were taken onboard the ISS and at NASA Ames Research Center (ground reference) during the experiment (see section “Materials and Methods” and Figure 2). The first photo session (photo session 1; Supplementary Figure 3) verified the successful activation of the experiment by an effective medium injection and consequent re-hydration of the samples (see section “Materials and Methods” for details). The post-fixation photo session (photo session 2, Figure 6) was performed after the experiment was completed, immediately following bacterial growth for 21 days and fixative injection. Despite providing qualitative rather than quantitative data, this approach allowed for the visualization of some features of the cell cultures that were no longer observable when the samples were retrieved on Earth after download. Aggregates were present in *S. desiccabilis* and *C. metallidurans* cultures in simulated Earth *g*. For *S. desiccabilis*, a smaller aggregate was observed in Mars *g* (Figure 6, yellow arrows) consistent with expectations from the theoretical calculations, but no aggregates were observed in *C. metallidurans* under Martian gravity condition. However, in the True-1*g* chambers, aggregates were absent or only produced in small amounts (i.e., *S. desiccabilis* and *C. metallidurans*). No aggregates were formed in any *B. subtilis* cultures.

In addition, it was possible to observe the presence of small gas inclusions (bubbles) in all samples exposed to microgravity (Figure 6, blue arrows). The bubbles in μ*g* appeared larger and more numerous in the cell cultures compared to the cell-free negative controls. In Mars *g*, few very small bubbles were present in the cell cultures, and none were observed in the negative controls. This suggested that their origin was biological. Bubbles were absent in 1 × *g* (Flight-1*g* and True-1*g*). The largest bubble visible in each container was caused by a void volume present in the hardware’s ducts.

## Discussion

In contrast to other organisms, the effects of reduced gravity regimens, such as microgravity or Martian gravity, on bacterial growth rates and final concentration are controversial. Some of the discrepancies could have been caused by the use of different experimental conditions, due to the variety of scientific questions addressed and the scarcity of spaceflight opportunities, as recently reviewed in Huang et al. (2018). The ESA BioRock experiment examined the microbe-mineral interactions of three bacteria with diverse characteristics (Supplementary Table 1), under identical growth conditions, in the same experimental apparatus and during the same spaceflight. The analysis of the liquid cultures after the spaceflight allowed us to measure the final cell concentration and optical density for the three bacterial cultures, and to test the hypothesis that the four examined gravity regimens would influence final cell concentrations.

We did not observe any significant differences in the final cell concentrations, within any of the three organisms, between the three gravity conditions in space (μ*g*, Mars *g* and Flight-1*g*) after 21 days (when all cultures had reached late stationary phase). This was an unexpected result. Due to the different physical and biological conditions, we expected to see significant differences between microgravity and gravities in which sedimentation occurred, i.e., 0.4 × *g* and 1 × *g* (Table 2). We suggest three potential explanations for the lack of significant differences observed.

One explanation could be that the cells were not affected by the physical conditions experienced and went through similar growth curves in all conditions, hence arriving at identical final concentrations in the late stationary phase. Arguing against it, lack of cell sedimentation occurring in microgravity is thought to have an important influence on cell growth (Benoit and Klaus, 2005), although difficult to predict (Klaus et al., 1997). Different authors suggested both positive and negative effects of lack of sedimentation in microgravity on bacterial cell growth (Marsh and Odum, 1979; Kacena et al., 1999a). For instance, lack of sedimentation in microgravity could influence continuous diffusional access to nutrients (Kacena et al., 1999a). Nutrient richness could influence the bacterial response to microgravity (Baker et al., 2004; Kim et al., 2013a, b). The theoretical calculations we presented in this study showed that sedimentation could occur in less than the total timeframe (21 days) of the experiment, along the entire length of the chamber, for both 1 × *g* and 0.4 × *g*, while sedimentation in microgravity conditions was estimated to be negligible in our experiment (Table 2). Our calculations showed that the cells would not be completely dispersed through the chamber by diffusion alone in 21 days, since the mean square distance traveled by a cell due to Brownian movement in this time was theoretically ∼1.3 mm^2^. Consistent with our calculations, no aggregation was observed in the BMRs subject to microgravity while they were visible in 1 × *g* and 0.4 × *g* (Figure 6), although aggregation by quorum sensing has been reported in microgravity (Mastroleo et al., 2013; Condori et al., 2016).

Motility should be considered. If sedimentation limited access to nutrients as reported above, motile bacteria would have had an advantage at a higher sedimentation rate as they could have escaped sedimentation. Some authors suggested that, in the absence of convective mixing (i.e., in μ*g*) or in the presence of sedimentation associated with gravity conditions on the Earth, motility is an advantage, as the movement of flagella allows cells to move into a fresh-nutrient area (Benoit and Klaus, 2007; Zea et al., 2017). Yet, the single non-motile microorganisms present in the BioRock experiment, *S. desiccabilis*, exhibited no obvious difference in final cell concentration in microgravity compared to higher gravities, similarly to the motile *B. subtilis* and *C. metallidurans*. Considering the arguments above, a complete lack of gravity-driven effects on bacterial growth is unlikely, and the first explanation provided is therefore implausible.

A second explanation could be that the conditions were indeed different between the gravity regimens, but the diverse factors exactly counteracted each other, leading to similar growth rates and final numbers within each bacterial species. For example, the increase in oxygen availability caused by sedimentation on the gas-semipermeable membrane might have been offset by a lack of cell access to nutrients, and these growth conditions also matched those under the microgravity diffusion-limited regime. However, a perfect counteraction of all the factors seems unlikely.

A third explanation is that, although the different physical environment in each gravity regime could have led to different growth rates and responses, by the end of the experiment all cells had effectively reached stationary phase, resulting in similar cell numbers within each bacterial species. Other space experiments reported comparable final cell concentrations with respect to their ground controls, despite differences in early growth phases (Klaus et al., 1997; Nicholson and Ricco, 2020), and after several days of growth in simulated microgravity compared to normal gravity control (Mastroleo et al., 2013). In our experiment, the bacterial cultures were grown for 21 days (which allowed us to maximize the bioleaching of elements from the rock). This is much longer than most space microbiology experiments, which tend to be on the order of hours or days. Our experiment would have allowed all the species to grow beyond exponential phase into stationary phase (similarly to the ground experiment in Figure 4), reaching similar final cell numbers in all conditions. Hence, we suggest the third hypothesis represents the most plausible explanation.

Although direct cell counts in the three organisms showed no significant difference between the different gravities in space, we did observe a significant optical density reduction in *S. desiccabilis* ground control (True 1-*g*) samples compared to samples in microgravity and simulated Mars gravity. Because optical density is influenced by cell shape and size, we cannot exclude that the results were due to a modified cell morphology in microgravity and Mars gravity, an effect shown in at least one other space experiment (Zea et al., 2017). Interestingly, these results were only observed when the optical densities of microgravity and Mars *g* were compared with True-1*g*, but not to simulated Earth *g* (Flight-1*g*) in space. Moreover, *B. subtilis* showed a significant difference in the final cell concentration (but not for optical densities) of Flight 1-*g* compared to True 1-*g*. Although the samples were treated with the same or very similar procedures and conditions (e.g., similar temperatures as verified using temperature logs), there are still unavoidable differences between spaceflight and ground experiments. For instance, in contrast to real Earth gravity, simulated Earth gravity achieved by centrifugation generates inertial shear and Coriolis forces (Van Loon et al., 2003; van Loon, 2015). The launch and download of spaceflight samples may have added differences such as higher gravity levels and vibration stresses (de Sousa et al., 2020). Although this occurred before and after the bacterial growth period, they may have conditioned sample preservation. In addition to different gravity, an object within a space capsule in LEO is exposed to a higher dose of cosmic radiations compared to an object on Earth (Horneck et al., 2010), however, we did not undertake a biochemical study of radiation effects in this experiment. Although we did not have empirically determined explanations for the discrepancies between simulated and true Earth gravities, they nevertheless showed that using simulated Earth gravity controls in space in addition to ground is important and can greatly improve the comparison and confidence in results.

In conclusion, the results reported here have demonstrated no statistically significant effect of gravity conditions on the final cell concentrations achieved by three microorganisms with different cell characteristics (with respect to motility, spore formation and growth rates), after 21 days of growth. This included simulated Martian gravity, to our knowledge the first experiment to report the effects of Martian gravity on bacteria using a space platform. From the human perspective, microorganisms could represent both enormous advantages and threats in space as on Earth. As the ambitions for future spaceflight missions expand, it is important to understand the long-term effects of low gravity on bacteria (Horneck et al., 2010). BioRock provided a rare example of a microbial growth experiment in space over multiple weeks, a timeframe that resembles those required in bio-industrial processes. Our data suggests that biotechnological applications such as bioproduction, bio-manufacture and life support systems on Mars will be possible, as final cell concentrations would not be deleteriously affected by Mars gravity under similar growth conditions reported here. It has been demonstrated that different partial gravities can lead to diverse results in some organisms, such as plants (Kiss et al., 2012; Kiss, 2014). However, we showed no difference on final cell concentration between microgravity and Mars gravity, suggesting that a similar result might be obtained in Moon and other partial gravities. Follow-up studies should be focused on repetition of the experiment, with the aim to obtain insights on the molecular mechanisms leading the bacterial growth of the microorganisms used. It would also be important to select further natural and artificial substrates for bacterial growth, as well as suitable and bioindustrially relevant microorganisms. Scaling-up the system needs to consider the effects of sedimentation and convective mixing and the specific physical and chemical parameters of a larger bioreactor. In addition to their impact on space exploration, our results could provide useful inputs to applications on Earth, such as bioproduction in space for terrestrial utilization (Benoit et al., 2006; Huang et al., 2018), or employment of controlled shear stress bioreactors in bioindustry (Kacena et al., 1999b; Lange et al., 2001; Jonczyk et al., 2013).

## Data Availability Statement

All datasets presented in this study are included in the article/Supplementary Material.

## Ethics Statement

Written informed consent was obtained from Luca Parmitano for the publication of any potentially identifiable images or data included in this article.

## Author Contributions

CSC conceived the BioRock experiment in the framework of ESA topical team. RS and CSC designed the experiments for this manuscript. RS, CSC, and ACW integrated the hardware for spaceflight and ground controls. RS, ACW, and WdW produced the experimental data. RM performed the spore enumeration. CSC and RS calculated the sedimentation rates. RS performed the data analyses. LP performed the procedures onboard the ISS with the supervision of BIOTESC. DLR (FMF, RM, and PR) and SCK CEN (RVH, IC, and NL) provided *B. subtilis* and *C. metallidurans* samples, respectively. RE and JW hosted the ground control experiment. Kayser Italia produced the hardware. ESTEC supervised the whole technical organization and BIOTESC supervised the flight procedures. RS wrote the manuscript. All authors discussed the results and commented on the manuscript, contributed to the article, and approved the submitted version.

## Conflict of Interest

VZ, MB, AM, SP, FC, and GL were employed by the company Kayser Italia S.r.l. The remaining authors declare that the research was conducted in the absence of any commercial or financial relationships that could be construed as a potential conflict of interest.
