# Supplementary material for: No Effect of Microgravity and Simulated Mars Gravity on Final Bacterial Cell Concentrations on the International Space Station: Applications to Space Bioproduction
Source: Front Microbiol. 2020 Oct 14;11:579156. doi: 10.3389/fmicb.2020.579156 (PMC7591705; doi:10.3389/fmicb.2020.579156)
Supplement: Supplementary file 1 [file Data_Sheet_1.pdf]

Supplementary Material

1.1 Supplementary Figures

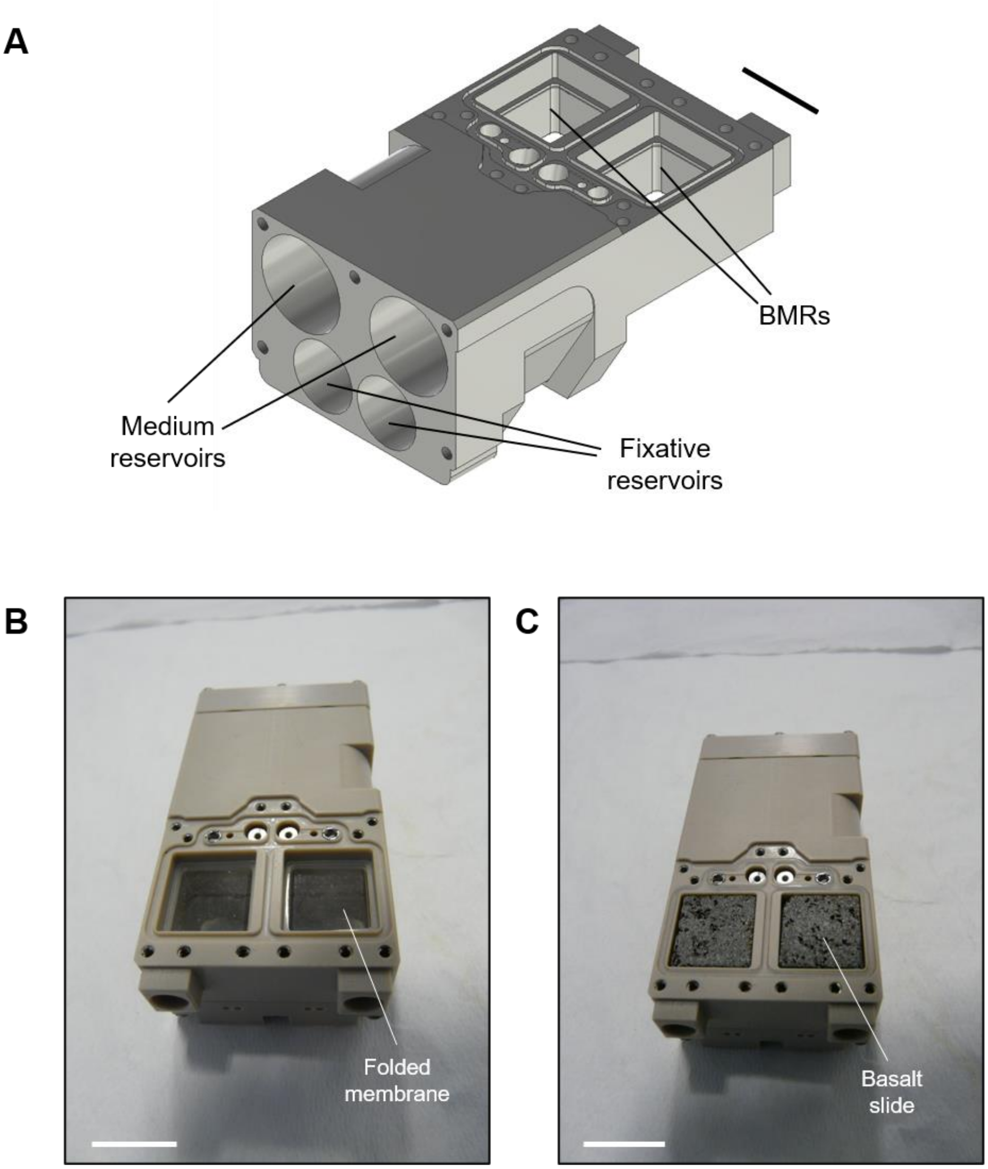

Supplementary Figure 1. The BioMining Reactor (BMR) hardware and Experimental Unit (EU) developed for the BioRock experiment. (A) Schematic illustration of the disassembled EU. The two medium reservoirs and two fixative reservoirs are visible. Each EU contains two BMRs with one culture chamber each. The portions that hold the basalt slides are visible. (B) Image of the hardware without the basalt slides. The folded transparent membrane is visible. (C) Image of the hardware locating one basalt slide in each BMR. The scale bars have been measured on the shorter side of the BMR and corresponds to ~14 mm.

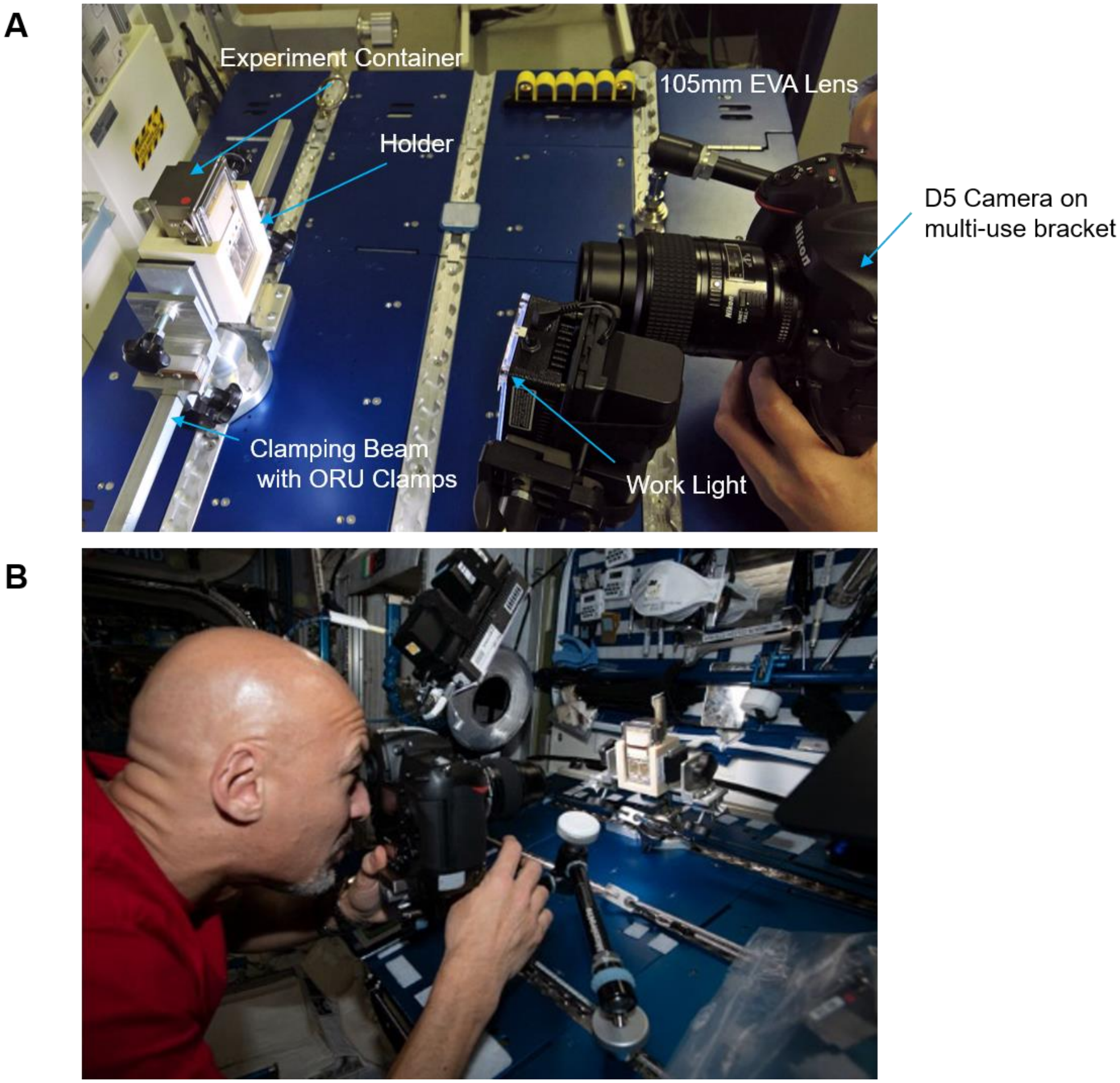

Supplementary Figure 2. Photo session setup (on ISS). (A) the Maintenance Work Area (MWA) that was used onboard the ISS to keep both the hardware and the camera in position during the photo session in microgravity; (B) astronaut Luca Parmitano is taking the photos with the help of the MWA onboard the ISS.

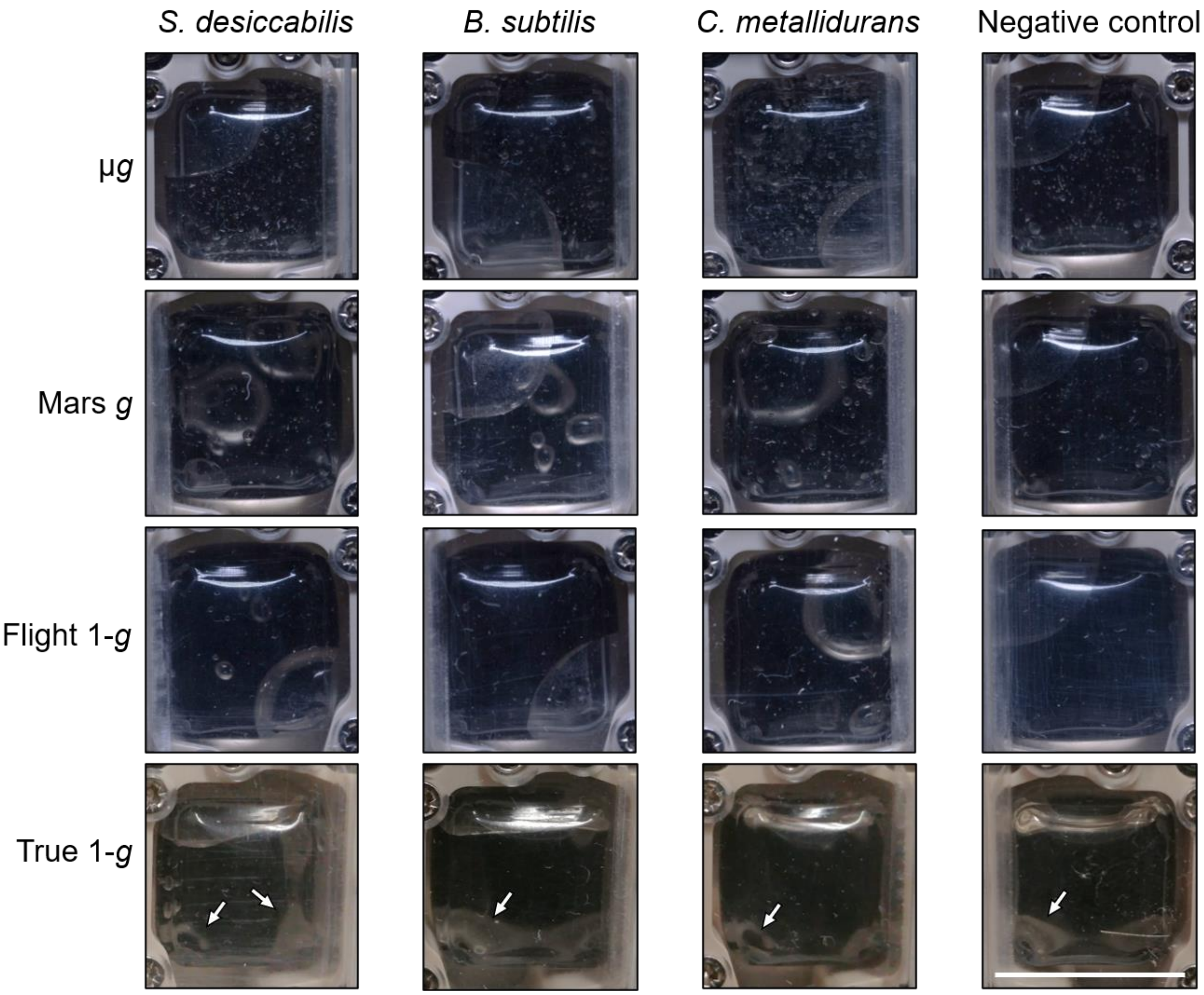

Supplementary Figure 3. Images from the first photo session (post-medium injection) onboard the ISS at the beginning of the experimental period. Representative photos of the individual culture chambers after medium injection and hardware activation. Each image shows one BMR. Pictures of the samples in microgravity (µ*g*), Mars gravity (Mars *g*) and terrestrial gravity (Flight 1-*g*) were taken during the spaceflight (in microgravity), while pictures of the ground controls (True 1-*g*) were taken at NASA Ames (at 1 x *g*). White arrows in the True 1-*g* samples indicate reflection of light from the internal parts of the culture chambers on the membrane. These features did not represent cell aggregates. The scale bar has been measured on the shorter side of the BMR and corresponds to ~14 mm.

1.2 Supplementary Tables

Supplementary Table 1. Summary of the main characteristics of the microorganisms used in the BioRock experiment.

**Species**	**Gram**	**Motility**	**Spore forming**	**Metabolism**	**Resistance to desiccation**	**Presence in rock/soil/metal rich environment**	**Previous involvement in space experiments**
Sphingomonas desiccabilis CP1D	-	-	-	Strictly aerobic [56]	Yes	Yes [55,56]	No
Bacillus subtilis NCIB 3610	+	+	+	Strictly aerobic in R2A 50% (but anaerobic growth is possible [62])	Yes	Yes [63,64]	Yes
Cupriavidus metallidurans CH34	-	+	-	Facultative aerobe [58], but strictly aerobe in R2A 50% (data not shown).	Yes, in appropriate conditions.	Yes [44,65]	Yes

Supplementary Table 2. Acceleration (gravity) measurements during the experiment on ISS in each of the Experimental Units. Values are indicated as mean±sd.

Condition	Content of EU	*g* acceleration measured (x *g*)
Microgravity	*S. desiccabilis* x 2	0.059±0.011
Microgravity	*B. subtilis* and control	0.054±0.028
Microgravity	*C. metallidurans* x 2	0.033±0.020
Microgravity	*S. desiccabilis* and control	0.064±0.036
Microgravity	*B. subtilis* x 2	0.043±0.024
Microgravity	*C. metallidurans* and control	0.053±0.024
Mars *g*	*S. desiccabilis* x 2	0.422±0.018
Mars *g*	*S. desiccabilis* and control	0.411±0.016
Mars *g*	*B. subtilis* x 2	0.461±0.017
Mars *g*	*B. subtilis* and control	0.437±0.017
Mars *g*	*C. metallidurans* x 2	0.470±0.018
Mars *g*	*C. metallidurans* and control	0.389±0.018
Flight 1-*g*	*S. desiccabilis* x 2	0.990±0.028
Flight 1-*g*	*S. desiccabilis* and control	1.047±0.024
Flight 1-*g*	*B. subtilis* x 2	1.039±0.036
Flight 1-*g*	*B. subtilis* and control	1.032±0.036
Flight 1-*g*	*C. metallidurans* x 2	1.036±0.027
Flight 1-*g*	*C. metallidurans* and control	1.027±0.025
